# Data on prevalence, distribution and risk factors for Foot and Mouth Disease in grazing cattle in haor areas of Bangladesh

**DOI:** 10.1016/j.dib.2019.104843

**Published:** 2019-11-23

**Authors:** Md. Shahidur Rahman Chowdhury, Md. Irtija Ahsan, Md. Jamal Khan, Md. Mahfujur Rahman, Md. Mukter Hossain, Ahmed Harun-Al-Rashid, Syed Sayeem Uddin Ahmed, Md Bashir Uddin

**Affiliations:** a Department of Medicine, Sylhet Agricultural University, Sylhet, 3100, Bangladesh; b Department of Epidemiology and Public Health, Sylhet Agricultural University, Sylhet, 3100, Bangladesh; c Upazila Veterinary Hospital, Sulla, Sunamganj, Bangladesh; d Department of Aquatic Resource Management, Sylhet Agricultural University, Sylhet, 3100, Bangladesh

**Keywords:** Cattle, FMD, Prevalence, Distribution, Risk factors, Haor areas, Bangladesh

## Abstract

Foot and mouth disease (FMD) is a highly contagious and devastating viral disease among all cloven-footed animals. In Bangladesh, the disease is endemic, with outbreaks occurring throughout the year in the haor regions. Thus, the FMD outbreaks impact livelihoods in the haor area and are of great concern. Therefore, a cross-sectional study was undertaken to evaluate the prevalence, distribution, and risk factors for clinical FMD in some selected areas of haor in Sylhet division of Bangladesh. We examined 1,388 cattle, of which 343 were clinically affected with FMD (prevalence 24.71%, CI95% = 22.44 – 26.98) during the period from July 2017 through June 2018. Though production loss was observed, no mortality was recorded in the infected animals. The data article shows the spatial distribution of FMD prevalence. The temporal pattern indicates a higher number of FMD cases in June (47.01%, CI95% = 38.97 – 55.07). The gender was found associated (OR = 2.98; p < 0.001) with the potential risk of FMD occurrence through univariate analysis. Besides, indigenous breeds of cattle (OR = 2.83; p < 0.001) are found to be more susceptible to FMD compared to exotic and crossbreeds. The risk factors identified in this article will serve as a baseline for the development of risk based FMD control program in future.

**Table:** Specifications Table

Subject	Veterinary Science and Veterinary Medicine
Specific subject area	An approach to disease control of grazing cattle in haor areas
Type of data	Graph, figure, table
How data were acquired	Data sets were obtained focusing on history, clinical (cardinal) signs for diagnosis of FMD. Moreover, data were collected through interviews with respondents from infected cattle raising by haor livelihoods.
Data format	Raw and analyzed
Parameters for data collection	The target population was cattle in the outbreak area. Cases of FMD were diagnosed entirely based on clinical signs [1,2]. Associations between FMD cases and risk factors were examined. Animal demography, spatial, and seasonal influences were considered for data collection.
Description of data collection	Investigation of clinical FMD in cattle was conducted at 10 villages from 4 unions of Sulla upazila from July, 2017 to June, 2018. The owners willingly visited the hospital with patients or veterinarian directly visited the owner's house to ensure the treatment of infected patients. Haor livelihoods were interviewed as well using a preformed questionnaire, and 1388 clinical cases of FMD data were documented in the record book.
Data source location	Upazila Veterinary Hospital, Upazila Livestock Office, Sulla upazila, Sunamganj, Bangladesh.
Data accessibility	All data are presented in this article including a supplementary file.
Related research article	D.M. Nyaguthii, B. Armson, P.M. Kitala, B. Sanz-Bernardo, A. Di Nardo, N.A. Lyons, Knowledge and risk factors for foot-and-mouth disease among small-scale dairy farmers in an endemic setting, Vet Res. 50 (2019) 1–12, http://doi:10.1186/s13567-019-0652-0 [3].

**Table:** 

**Value of the Data** • The data provides information on epidemiological indices of FMD in haor areas. • The data on the prevalence and distribution of foot and mouth disease (FMD) will be useful for future prevention and control of FMD at Sulla upazila in Sunamganj district, Bangladesh. • The data indicate unique features of FMD infection in grazing cattle that might inform a monitoring plan for local disease control. Moreover, understanding of these risk factors will assist developing a risk-based control strategy for haor areas.

## 1 Data

The presented data show the prevalence, spatial and temporal distribution of Foot and Mouth Disease (FMD) in grazing cattle of haor areas of Bangladesh. Also, the data shows the significance of animal demography (age, sex, and breed) and univariate analysis of risk factors of FMD in grazing cattle. Along with the existing measures drafted and practiced by the Department of Livestock Services, Bangladesh [4,5] to control the FMD, the data on the risk of FMD and associated control measures including movement restriction, vaccination, and a disease monitoring plan will be helpful for local disease control. Therefore, this data will be useful for improving the current measures and generating new control measures against the FMD in this area and will work as a reference for areas with similar geographical features.

### 1.1 **Prevalence of FMD and spatial distribution**

**Fig. 1:**
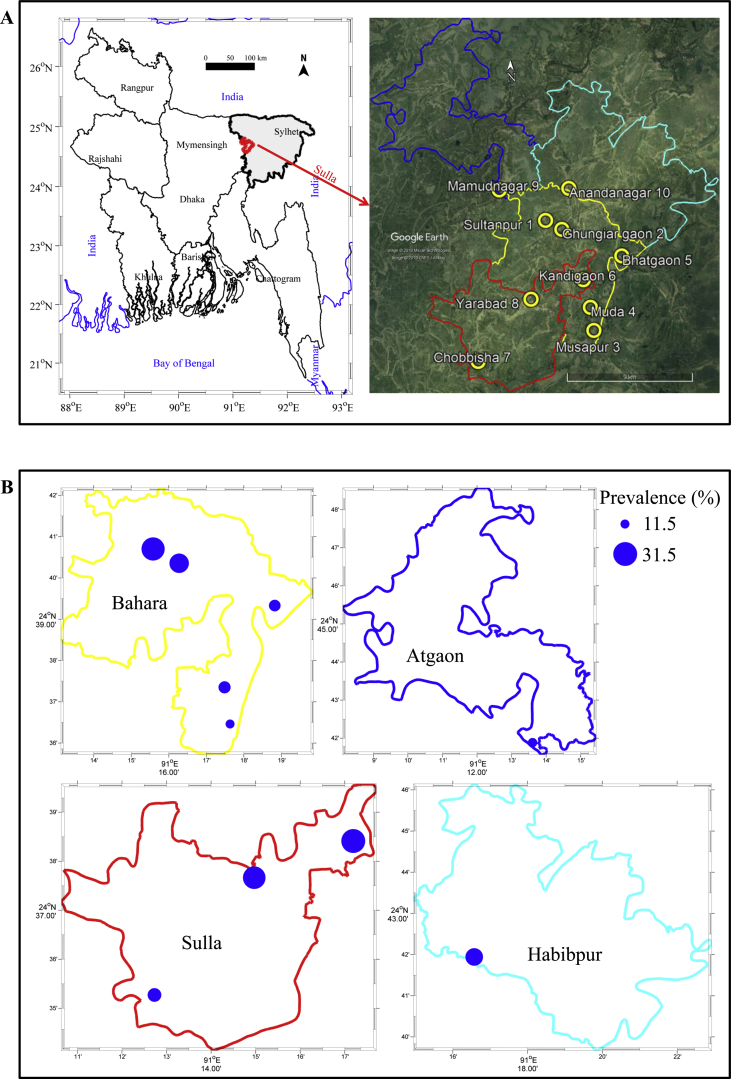
A & B: Maps illustrating the (A) study area with location of 10 villages, and (B) village-wise prevalence (%).

The prevalence was measured to estimate the disease burden in the study villages. The overall prevalence of clinical FMD in cattle was 24.71% (N=1388). The estimated prevalence of clinical FMD in cattle at different villages was 30.13% in Sultanpur, 25.26% in Ghungiargaon, 11.50% in Musapur, 15.63% in Meda, 14.88% in Bhatgaon, 31.14% in Kandigaon, 17.65% in Chobbisha, 29.41% in Yarabad, 22.73% in Anandanagar, and 19.33% in Mamudnagar (Fig. 1, A and B; Supplementary Table S1). The locations of the infected cattle were spatially identified based on the surrounding landmarks like a mosque, school, college, markets, etc. via Google Earth Pro®. Geographical coordinates of centroids of the respective affected areas in the village were plotted on a union level map (Fig. 1). Thus, the maps graphically represent the information about the village location of the FMD outbreak, including prevalence estimate.

### 1.2 Temporal distribution of FMD

**Fig. 2:**
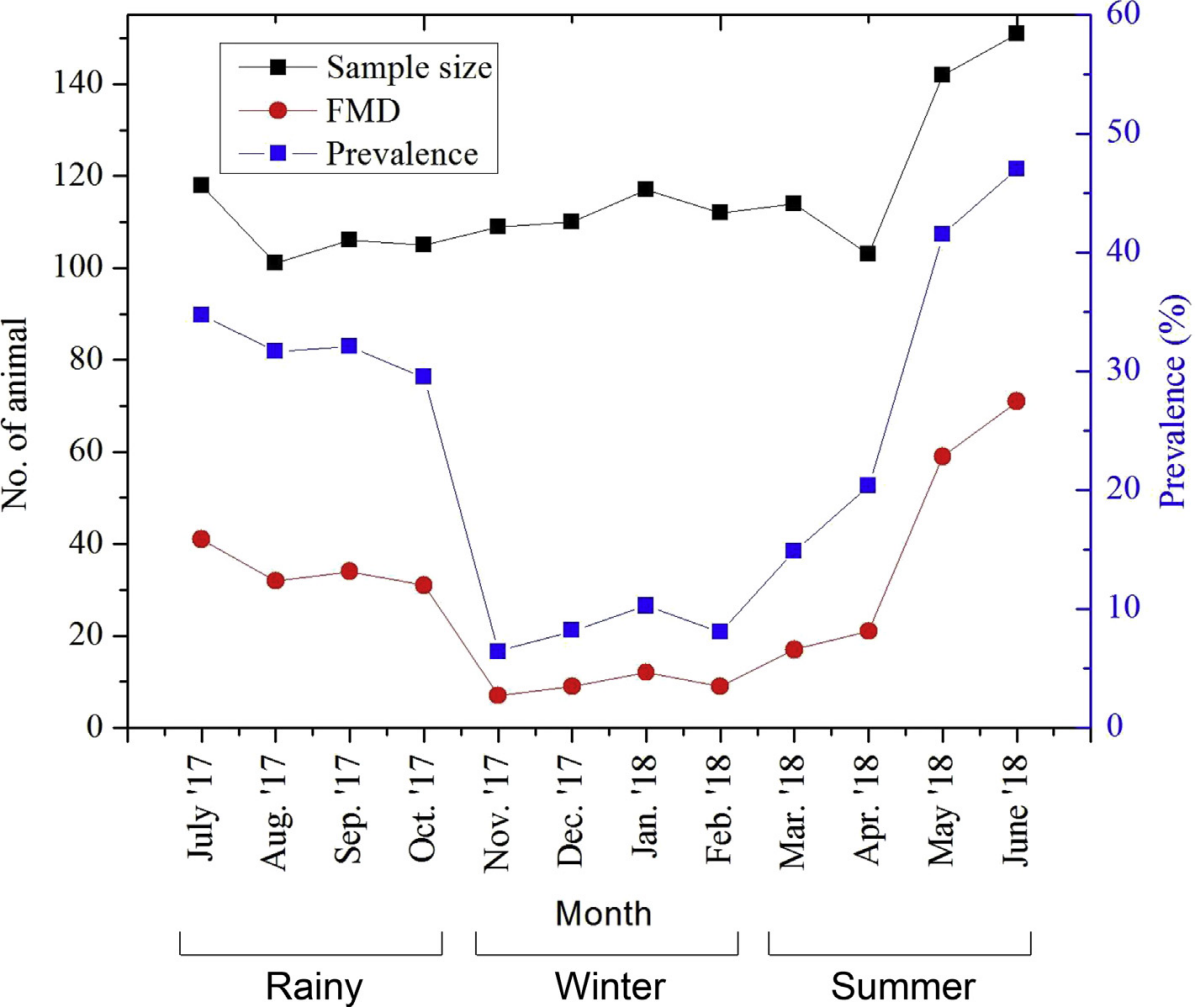
Temporal distribution of FMD cases officially recorded in Upazila Livestock Office, Sulla. This graph illustrates the monthly prevalence against FMD from July 2017 to June 2018.

Analysis of temporal data shows that the outbreaks activity was high during July- October 2017 and May-June 2018, over the one year study period (Fig. 2). The highest number of outbreaks occurred in June 2018, whereas the lowest quantity of outbreaks occurred in November 2017. Usually, in the study areas, harvesting time for paddy is from March through April. Free grazing of cattle in the paddy fields after harvesting is a common practice in Bangladesh. Thus, June is the peak time for free grazing when animals share the common grazing lands and nearby watering points with neighboring villages. This free grazing hastens the horizontal spreading of infection from one cattle to another [4,6]. Moreover, high wind flow during that period might also contribute to the spread of FMD virus [7]. The analysis showed that outbreaks were around four times higher in the summer season [32.94% (CI95%=28.85 – 37.03)] than in the winter season [8.26% (CI95%=5.70 – 10.82)] [Supplementary Table S2]. No visible outbreaks activity was evident in the winter season. Overall, the distribution of outbreaks was similar in the summer and rainy season.

### 1.3 **Factors associated with the occurrence of FMD and univariable analysis**

**Fig. 3:**
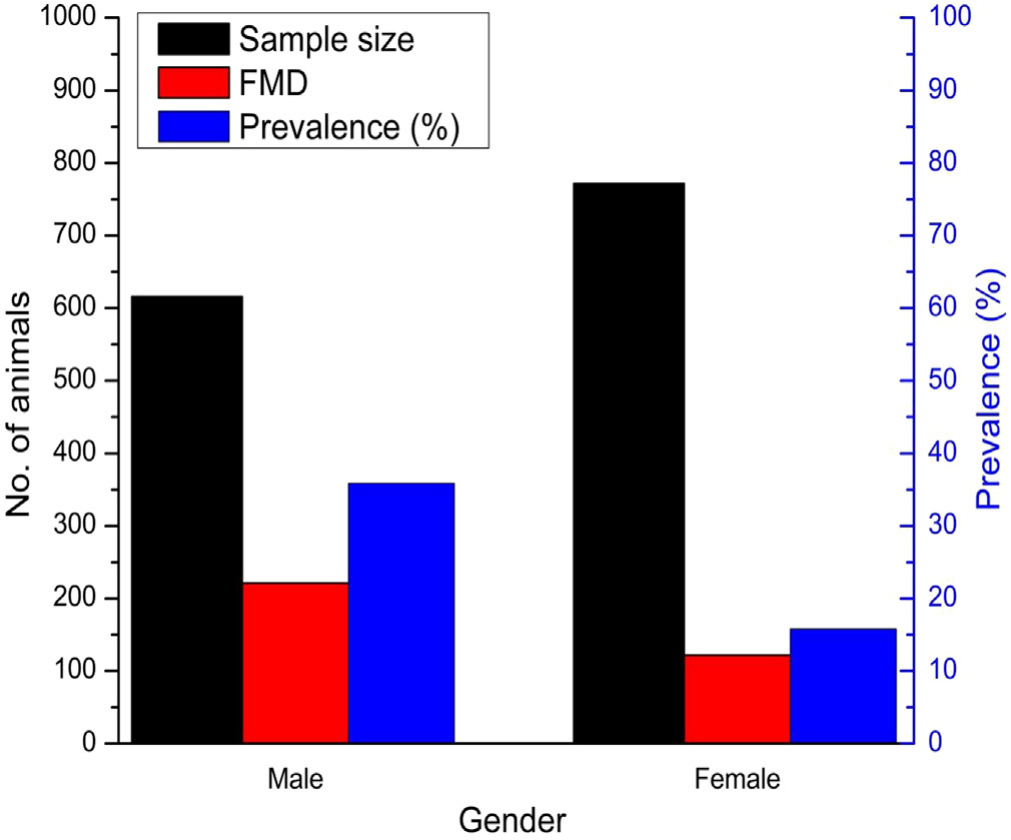
Sex-wise prevalence (%) of FMD cases.

**Fig. 4:**
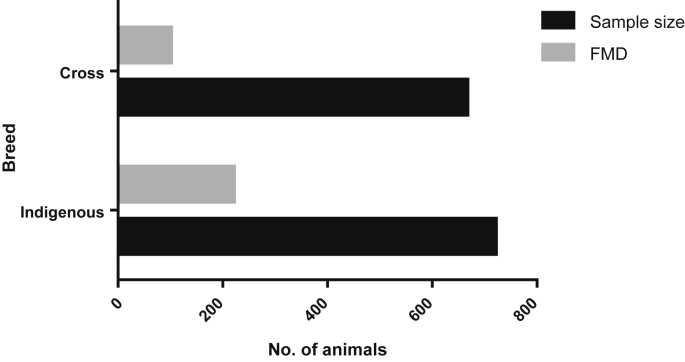
Breed-wise FMD cases.

**Fig. 5:**
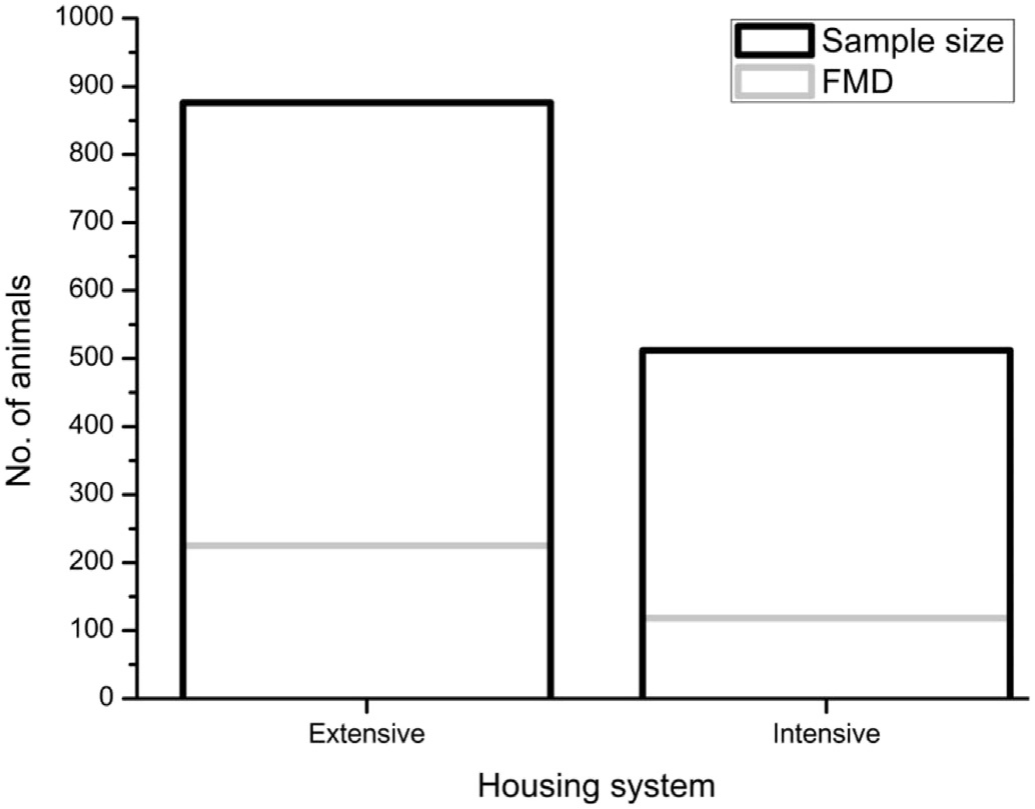
Housing system - wise prevalence (%) of FMD cases.

The frequency of FMD cases in old cattle (>4years) was 36.23% (CI95%=32.57 – 39.88), higher than the younger (<4 years) cattle (Table 1). Male cattle [35.88% (CI95%=32.08 – 39.67)] were more commonly infected with FMD than female cattle [15.80% (CI95%=13.22 – 18.38]. Based on the univariable analysis, the present study identified some factors to be associated with an increased risk of clinical FMD cases. The factors are gender (gender (sex-wise distribution, Fig. 3, Supplementary Table S3), breed (breed-wise distribution, Fig. 4, Supplementary Table S4). The risk of getting FMD cases in male cattle was 2.98 times higher (OR = 2.98; p < 0.001) than female cattle. An earlier study reported that FMD infection is more common in younger animals and male sex group than the older and female animals, respectively [6]. Male cattle are usually used for draught purposes, which enhance the possibility of skin damages that might lead to higher susceptibility of FMD [7]. Based on the univariable analysis (Table 2), the indigenous cattle were found to be significantly associated with the FMD [OR=2.83, CI95%=2.19 – 3.68, p = < 0.001] compared to crossbreed cattle. We examined the influence of intensive and extensive housing system on the risk of FMD cases (housing system-wise distribution, Fig. 5, Supplementary Table S5); however, did not found any significant association [OR=1.15, CI95%=0.89 – 1.48; p = 0.271].

**Table 1:** Age wise prevalence (%) of FMD in cattle.

Age category	Animals examined	FMD	Prevalence (CI_95%_)
Young (<2 yr)	396	31	7.83 (5.17–10.49)^a^
Adult (2–4 yr)	324	70	21.60 (17.10–26.11)^b^
Old (>4 yr)	668	242	36.23 (32.57–39.88)^c^

^**a,b,c**^ Age with different superscript vary significantly in prevalence (*p* < 0.05).

**Table 2:** Univariable analysis of risk factors of FMD in cattle.

Variables	Animals examined (%)	FMD (%)	Odds Ratio (CI_95%_)	P-value
**Gender**				<0.001
Male	616 (44.38)	221 (35.88)	2.98 (2.31–3.84)	
Female	772 (55.62)	122 (15.80)	1	
**Breed**				<0.001
Indigenous	721 (51.95)	242 (33.56)	2.83 (2.19–3.68)	
Cross	667 (48.05)	101 (15.14)	1	
**Housing system**				0.271
Extensive	876 (63.11)	225 (25.68)	1.15 (0.89–1.48)	
Intensive	512 (36.89)	118 (23.05)	1	

**chi-square (χ**^**2**^**) test:***p*-value <0.05 considered as significance.

## 2 Experimental design, materials, and methods

### 2.1 Study area

This study was performed in Sulla upazila, Sunamganj, Bangladesh. Sulla upazila, a region of 260.74 km^2^, is located in between 24°34′ and 24°49′ north latitudes and in between 91°08′ and 91°23′ east longitudes surrounded by haor or beels. It is comprised of four local government areas (unions) *viz*. Atgaon, Bahara, Sulla, and Habibpur. From these four unions a total of 10 villages were included in this study where FMD was perceived to be endemic as recorded by the Upazila Livestock Office, Sulla, Sunamganj, Bangladesh. These areas are characterized by diverse agro-ecological zones progressively varying in climates. Livestock husbandry is mainly characterized by extensive farming system in which animals are allowed to graze freely during day time and housed only during night time. Cattle population of the zones was estimated at 32,884 in Sulla [Upazila Livestock Office, Sulla; personal communication].

### 2.2 Study population and data collection

The present study was conducted to determine the prevalence, describe the spatial and temporal distribution, and to identify the risk factors associated with FMD cases in the study areas. The target population was haor cattle in the outbreak area. Investigation of clinical FMD in cattle was conducted from July 2017 through June 2018. A total number of 1,388 clinical cases of cattle were registered at the Upazila veterinary hospital. The owners of the affected animals willingly visited the hospital with patients for treatment, or a veterinarian visited the owner's house to ensure the treatment of infected patients. Information on livelihoods of the haor area was collected through interviews, using a pretested questionnaire, and the data were recorded in a record book. The study examined the plausible factors associated with the FMD infection for each animal regardless of animal demography (age, sex, and breed), seasonal or more specifically monthly influences, etc. The information on housing types, such as intensive and extensive, were also collected. Cases of FMD were diagnosed entirely based on clinical signs [1,2] and clinical history after a physical examination. Clinical cases that resembled FMD (e.g., vesicular stomatitis, rabies, and foot rot) were also recorded. Differential diagnosis was made between FMD and resembled cases based on clinical signs and lesions. FMD was differentiated from vesicular stomatitis, which produces only oral lesions only; Rabies shall have dog bite history and frenzy or drowsiness; foot rot does not have oral lesions, but vesicle formation in the interdigital cleft is seen [2]. The patients' conditions and recovery, as well as documentation of incomplete information, were followed up by subsequent visits or cell phone interviews. The collected data were later validated using information documented by the Upazila Livestock Office, Sulla, Sunamganj, Bangladesh.

### 2.3 Data analysis

The infected village location of each union was geographically visualized in maps by using MATLAB®. The collected data were summarized by using a Microsoft Excel® data spreadsheet. Statistical analysis was performed by using Statistical Package for the Social Sciences (SPSS^TM^) version 21.0 (IBM Corp., Armonk, NY, USA). Associations between FMD cases and associated risk factors were estimated in terms of odds ratio with 95% confidence intervals. A *p*-value <0.05 was considered as an indicator of statistical significance. Graphs were generated using GraphPad Prism 6.

### 2.4 Limitations of the study

The data in this study is based on reported cases diagnosed through cardinal clinical signs only. In the absence of laboratory tests carried out for the registered cases, differential diagnosis with FMD cases was made for other diseases having similar clinical signs. Only cattle cases are included in this study. However, FMD cases of other ruminant species would worthy to investigate. Thus, we suggest future research that will include other ruminant species of the haor area.
