# Supplementary material for: Data on prevalence, distribution and risk factors for Foot and Mouth Disease in grazing cattle in haor areas of Bangladesh
Source: Data Brief. 2019 Nov 23;28:104843. doi: 10.1016/j.dib.2019.104843 (PMC6962694; doi:10.1016/j.dib.2019.104843)
Supplement: Multimedia component 2 [file mmc2.docx]

**Supplementary File_FMD**

**Supplementary Table S1: Village wise proportionate prevalence (%) of FMD**

| **Union** | **Village** | **Animals Examined** | **FMD** | **Prevalence (%)** |
| --- | --- | --- | --- | --- |
| Bahara | Sultanpur | 531 | 160 | 30.13 |
|  | Ghungiargaon | 194 | 49 | 25.26 |
|  | Musapur | 87 | 10 | 11.50 |
|  | Meda | 96 | 15 | 15.63 |
|  | Bhatgaon | 121 | 18 | 14.88 |
| Sulla | Kandigaon | 167 | 52 | 31.14 |
|  | Chobbisha | 34 | 6 | 17.65 |
|  | Yarabad | 17 | 5 | 29.41 |
| Habibpur | Anandanagar | 22 | 5 | 22.73 |
| Atgaon | Mamudnagar | 119 | 23 | 19.33 |

**Supplementary Table S2: Season wise prevalence of FMD in cattle**

| **Name of the Season** | **Name of the month** | **No. of cattle examined** | **No. of FMD positive cattle** | **Prevalence (Confidence Interval) (%)** |
| --- | --- | --- | --- | --- |
| **Rainy** | July, 2017 | 118 | 41 | 34.75 (26.03 – 43.46) |
|  | August, 2017 | 101 | 32 | 31.68 (22.45 – 40.91) |
|  | September, 2017 | 106 | 34 | 32.08 (23.04 – 41.11) |
|  | October, 2017 | 105 | 31 | 29.52 (20.65 – 38.39) |
|  | **Sub-total** | **430** | **138** | **32.09 (27.66 – 36.52)^a^** |
| **Winter** | November, 2017 | 109 | 7 | 6.42 (1.75 – 11.10) |
|  | December, 2017 | 110 | 9 | 8.18 (2.98 – 13.39) |
|  | January,2018 | 117 | 12 | 10.26 (4.68 – 15.84) |
|  | February,2018 | 112 | 9 | 8.04 (2.92 – 13.15) |
|  | **Sub-total** | **448** | **37** | **8.26 (5.70 – 10.82)^b^** |
| **Summer** | March,2018 | 114 | 17 | 14.91 (8.27 – 21.55) |
|  | April,2018 | 103 | 21 | 20.39 (12.48 – 28.30) |
|  | May,2018 | 142 | 59 | 41.55 (33.34 – 49.75) |
|  | June,2018 | 151 | 71 | 47.02 (38.97 – 55.07) |
|  | **Sub-total** | **510** | **168** | **32.94 (28.85 – 37.03)^c^** |
|  | **Total (Overall)** | **1388** | **343** | **24.71 (22.44 – 26.98)** |

**^a,b,c^ Seasons with different superscript vary significantly in prevalence (p<0.05)**

**Supplementary Table S3: Gender wise prevalence of FMD in cattle**

| **Gender** | **No. of cattle examined** | **No. of FMD positive cattle** | **Prevalence (Confidence Interval) (%)** |
| --- | --- | --- | --- |
| Male | 616 | 221 | 35.88 (32.08 – 39.67)**^a^** |
| Female | 772 | 122 | 15.80 (13.22 – 18.38)**^b^** |

**^a,b^ Gender with different superscript vary significantly in prevalence (p<0.05)**

**Supplementary Table S4: Breed wise prevalence of FMD in cattle**

| **Breed** | **No. of cattle examined** | **No. of FMD positive cattle** | **Prevalence (Confidence Interval) (%)** |
| --- | --- | --- | --- |
| Indigenous | 721 | 242 | 33.56 (30.11 – 37.02)**^a^** |
| Cross | 667 | 101 | 15.14 (12.42 – 17.87)**^b^** |

**^a,b^ Breed with different superscript vary significantly in prevalence (p<0.05)**

**Supplementary Table S5: Housing system wise prevalence of FMD in cattle**

| **Housing system** | **No. of cattle examined** | **No. of FMD positive cattle** | **Prevalence (Confidence Interval) (%)** |
| --- | --- | --- | --- |
| Extensive | 876 | 225 | 25.68 (22.79 – 28.58)**^a^** |
| Intensive | 512 | 118 | 23.05 (19.39 – 26.71)**^b^** |

**^a,b^ Housing system with different superscript vary significantly in prevalence (p<0.05)**
